# Uncovering a Complex Virome Associated with the Cacao Pathogens *Ceratocystis cacaofunesta* and *Ceratocystis fimbriata*

**DOI:** 10.3390/pathogens12020287

**Published:** 2023-02-09

**Authors:** Roy Bogardid Ardón Espinal, Sabrina Ferreira de Santana, Vinícius Castro Santos, Gabriela Nicolle Ramos Lizardo, Raner José Santana Silva, Ronan Xavier Corrêa, Leandro Lopes Loguercio, Aristóteles Góes-Neto, Carlos Priminho Pirovani, Paula Luize Camargos Fonseca, Eric Roberto Guimarães Rocha Aguiar

**Affiliations:** 1 Center for Biotechnology and Genetics, Universidade Estadual de Santa Cruz, Ilhéus 45662-900, Brazil; 2 Department of Microbiology, Universidade Federal da Bahia, Salvador 40170-115, Brazil; 3 Department of Biochemistry and Immunology, Universidade Federal de Minas Gerais, Belo Horizonte 31270-901, Brazil

**Keywords:** mycovirus, phytopathogen, virus, fungi, RNA interference

## Abstract

*Theobroma cacao* is one of the main crops of economic importance in the world as the source of raw material for producing chocolate and derivatives. The crop is the main source of income for thousands of small farmers, who produce more than 80% of the world’s cocoa supply. However, the emergence, re-emergence and proliferation of pathogens, such as *Ceratocystis* spp., the causative agent of *Ceratocystis* wilt disease and canker disease, have been affecting the sustainability of many crops. Fungal control is laborious, often depending on fungicides that are expensive and/or toxic to humans, prompting researchers to look for new solutions to counteract the proliferation of these pathogens, including the use of biological agents such as mycoviruses. In this study, we investigated the diversity of microorganisms associated with the *T. cacao* pathogens *Ceratocystis cacaofunesta* and *Ceratocystis fimbriata* with a focus on the virome using RNA sequencing data available in public databases. We used a comprehensive bioinformatics pipeline containing several steps for viral sequence enrichment and took advantage of an integrated assembly step composed of different assemblers followed by sequence similarity searches using NCBI nonredundant databases. Our strategy was able to identify four putative *C. cacaofunesta* viruses (hypovirus, sclerotimonavirus, alphapartitivirus and narnavirus) and six *C. fimbriata* viruses (three alphaendornaviruses, one victorivirus and two mitoviruses). All the viral sequences identified showed similarity to viral genomes in public databases only at the amino acid level, likely representing new viral species. Of note, we present the first report of viruses associated with the cacao pathogens *C. cacaofunesta* and *C. fimbriata* and the second report of viral species infecting members of the *Ceratocystidaceae* family. Our findings highlight the need for further prospective studies to uncover the real diversity of fungus-infecting viruses that can contribute to the development of new management strategies.

## 1. Introduction

*Theobroma cacao* L. is one of the most important cash crops in many tropical regions of the world, with planted areas comprising ~8.2 million ha [1]. The nuts are used to make chocolate and cosmetics [2]. The cacao crop is the main source of income for millions of small farmers in many countries (e.g., Ivory Coast, Ghana, Ecuador and Brazil), who account for more than 80% of the global supply of cocoa [3,4]. Overall, worldwide food security is threatened by the effects of biotic and abiotic stresses on crops, with environmental (mostly climatic) and genetic factors being the main causes of new pathogens and insect pests. Under these circumstances, a major challenge for plant breeders is the generation and improvement of resistant crops [5], since phytopathogens can reduce yields by up to 20% or more [6].

*Ceratocystis* spp. (*Ascomycota* phylum, *Sordariomycetes* class) include aggressive pathogenic fungal species, which attack an array of economically important plant species worldwide. These pathogens cause the diseases Ceratocystis wilt and canker, which often kill the host plants, causing significant losses to farmers and jeopardizing the sustainability of agricultural production [7]. In the last two decades, emerging *Ceratocystis* diseases have increased considerably in Central and South America [7,8]; *Theobroma cacao* L. and *Eucalyptus* sp. can be infected by *Ceratocystis cacaofunesta* and *C. fimbriata*, respectively, which reduces the productive capacity of these plants [9]. The causal agent of *Ceratocystis* wilt of cacao (CWC, or ‘machete disease’) [10] is one of the three well-established host-specific pathogens in the genus [7], causing disease only to cacao plants [11]. Besides pruning, another transmission mechanism of this pathogen is through contact of the plant with different insect vectors, such as those of the *Xyleborus* genus (*Coleoptera-Scolytidae*) [10,12,13]. This fungus penetrates the plant through holes and wounds. The CWC pathogenesis is characterized by irreversible damage to the plant’s vascular system, leading to death on the order of 10^4^ to 10^5^ individuals per plantation, hence significantly reducing cocoa production per area [7,14]. This disease poses a serious threat to global cocoa production due to the high risk of its spread [7,15] by human activities, the main propagation mode described [3].

The control of this fungal pathogen is difficult, so the search for alternative economically viable and environmentally friendly solutions is necessary [14]. Previous research has described the potential of controlling oak wilt (caused by *C. fimbriata*) by using the bacterium *Pseudomonas chlororaphis* subsp. *aureofaciens*, based on the synthesis of volatile compounds by SPS-41, which showed significant inhibitory effects on mycelial growth and spore germination of *C. fimbriata* [16]. In terms of cacao (a perennial plant), one of the most efficient strategies to minimize losses is the selection of resistant varieties. However, the substitution of existing genotypes with resistant genotypes on plantations is a slow process. In addition, the various cacao pathogens and their specific modes of pathogenicity hamper the selection of plants simultaneously resistant to all of them [2]. Each pathogen affects different organs of the plant, e.g., *Moniliophthora perniciosa* in vegetative shoots, pods, and floral cushions [17]; *Phytophthora* spp. in pods and leaves; [18] and *Ceratocystis cacaofunesta* in the vascular system [11]. Finally, the use of biocontrol agents (BCAs) such as *Trichoderma* spp. has proven to be effective in inhibiting the in vitro growth and reducing spore germination of *C. cacaofunesta* [19]. The antifungal tebuconazole present in the medicinal plant *Adiantum latifolium* also had an important inhibition effect on mycelial growth and propagation of *C. cacaofunesta*, mainly in places where genetic selection and proper handling of instruments at the time of pruning fail altogether [14].

Among the BCAs, mycoviruses are good candidates, since they have been found to infect all major taxonomic groups of fungi, including phytopathogenic species [20]. Several mycoviruses cause abnormal symptoms in the host, such as hypovirulence and wasting [21]. A successful example of a viral BCA against phytopathogenic fungi is *Cryphonectria hypovirus* 1 (CHV1), which causes hypovirulence in *Cryphonectria parasitica* and has been used to reduce infection of figs [22]. Similarly, *Fusarium oxysporum alternavirus* 1 (FoAV1) was recently found to reduce the fungal growth and symptoms of *Fusarium* wilt (a vascular disease) in *Lilium brownii* [23]. For phytopathogenic fungi such as *Sclerotinia sclerotiorum*, *S. nivalis*, *S. minor* and *Botrytis cinerea*, *Sclerotinia sclerotiorum partitivirus* 1 can affect the virulence of the host fungus on the plant [24]. Thus, these and other mycovirus species are very promising as biological control agents against fungal diseases [21,25,26].

Fungi have developed different defense mechanisms against their own pathogens, including viruses. The conserved defense mechanisms based on RNA interference (RNAi) viral silencing have been studied in many fungal species, including *Cryponectria parasitica*, *Aspergillus nidulans* and *Rosellinia necatrix* [27,28]. These mechanisms act against invading nucleic acids, such as those produced by viruses, transposons or transgenes, in a wide spectrum of fungal species [26,29,30,31]. In fungi, the different RNAi pathways are related to three main functions: genomic defense, heterochromatin formation and gene regulation [32,33,34,35]. Although most fungal species possess the RNAi mechanism, there are several filamentous fungi (e.g., *Candida albicans* and *Ustilago maydis* [27,36]) and yeasts (e.g., *Saccharomyces cerevisiae* [32]) that seem to lack all or most of the components necessary for RNA silencing to occur [27,36]. This means that the RNAi pathway is not required by some unicellular organisms [32]. Fungal viruses are less explored than plant viruses, therefore their real diversity still unknown. Similarly, regarding plant viruses, most mycoviruses have been studied in the context of controlling fungal phytopathogens [37]. The application of bioinformatics and next-generation sequencing technologies (NGS) has contributed greatly to the discovery of new viruses in many organisms, including fungi [26]. NGS techniques have been used to detect the presence of viral sequences, regardless of the viral titer of the sample, since this method does not require prior knowledge of the genomic sequences of the candidate viruses [26,37,38].

We decided to investigate the virome associated with the important *T. cacao* pathogens *C. cacaofunesta* and *C. fimbriata* using public RNA sequencing data. Our bioinformatics strategy led to the identification of many viruses in both *Ceratocysitis* species, including members of *Hypoviridae*, *Alphaendornaviridae* and *Alphapartitiviridae* families that have been described as containing viruses with biotechnological potential. Of note, this is the first report of viruses associated with the cacao pathogens *C. cacaofunesta* and *C. fimbriata* and the second report of viruses infecting members of the *Ceratocystidaceae* family. Our findings highlight the need for new studies to uncover the real diversity of mycoviruses that can contribute to the development of new biofungicides.

## 2. Materials and Methods

### 2.1. Acquisition of RNA Libraries

*Ceratocystis cacaofunesta* and *Ceratocystis fimbriata* public RNA-seq libraries were downloaded from the Sequence Read Archive (SRA) database from NCBI. We used two libraries: one from *C. cacaofunesta* (SRR6217952) constructed from a medium enriched with cacao xylem and deeply sequenced using the Illumina HiSeq 2500 Platform with a single layout [10] and one from *C. fimbriata* isolate CBS 114,723 (SRR8599076) constructed from a culture maintained at the Forestry and Agricultural Biotechnology Institute (FABI) of the University of Pretoria, which was deeply sequenced using the Ion Torrent Proton instrument, also with a single layout [39,40].

### 2.2. Bioinformatic Analysis

#### 2.2.1. Transcriptome Assembly

Public RNA-seq libraries were preprocessed using the tools FastQC (version 0.11.9) [41] and Trim Galore (version 0.6.7 + galaxy0) [42]. Removal of host-derived sequences was performed by aligning preprocessed reads against each specific reference genome—*C. cacaofunesta* (PEJQ01000008.1) and *C. fimbriata* (VNIJ01000001.1)—using Bowtie2 (version 2.4.5 + galaxy1) [43] with default settings. The unmapped reads were used in the assembly step, taking advantage of different assemblers: SPAdes (version 3.15.4 + galaxy1) [44], rnaviralSPAdes (version 3.15.4 + galaxy1) [45] and Trinity (version 2.9.1 + galaxy2) [46], with default settings. The assembled transcripts derived from SPAdes, rnaviralSPAdes and Trinity were used as input for CAP3 (version 10.2011) [47] in order to consolidate the results and extend possible viral sequences.

#### 2.2.2. Identification of Viral Sequences

The viral contigs were identified by sequence similarity search through the Diamond (version 2.0.15 + galaxy0) [48] with BlastX mode using the viral Refseq from NCBI as the database. Statistical analysis of assembled contigs was performed with Fasta Statistics (version 2.0) [49] (Supplementary Table S1). All tools and programs used can be found online on the Galaxy Australia platform [50].

#### 2.2.3. Manual Curation of Viral Genomes

Non-retroviral sequences were filtered by length >500 nt and checked by manual inspection including online Blast searches, structural annotation, and analysis of conserved domains. Nonredundant contigs were subjected to sequence similarity searches using the online Basic Local Alignment Search Tool [51] at the nucleotide and amino acid levels. Sequences identified with coverage greater than 30% with known species and E-values < 1 × 10^−3^ for protein and <1 × 10^−5^ for nucleotide were further analyzed (Supplementary Table S2). Representative viral sequences from each group were selected, classified according to the highest bit score and used in subsequent analyses. Structural annotation regarding predicted open reading frames (ORFs) was carried out using the online tool ORFfinder [52] except for viruses related to the *Mitoviridae* and *Narnaviridae* families, for which genetic code 4 was used (Mold, Mitochondrial Protozoa and Coelenterates and Mycoplasma/Spiroplasma), with acceptance of “ATG” and alternative initiation codons. The ORFs were used for the identification of conserved protein domains as well as homologous superfamilies of the different amino acids of replication-associated proteins in the viruses *C. cacaofunesta* and *C. fimbriata* using the online tools InterProScan [53] and HMMER [54,55,56].

#### 2.2.4. Integrative Genome Assembly

In order to improve possible viral genomes, an integrative assembly strategy was also performed running the different tools rnaviralSPAdes [45], metaSPAdes [57], Oases [58], Metavelvet [59] and IDBA_UB [60]. The results were consolidated using CD-HIT [61] to join sequences with more than 90% similarity. The assembled sequences were manually investigated and used along with raw read alignment to correct misassembled regions. Manually curated viral sequences identified in our work were deposited in the NCBI GenBank Third Party Annotation database under accession numbers BK062942-BK062946, BK063053-BK063059, and can also be found in the Supplementary Data.

### 2.3. Phylogenetic Analysis

Viral sequences that presented similarity to polymerase or polyprotein-coding genes were used in phylogenetic analysis. Datasets composed of public protein sequences (Supplementary Table S3) related to *Hypoviridae*, *Fusariviridae*, *Nyamiviridae*, *Mymonaviridae*, *Potyviridae*, *Partitiviridae*, *Fiersviridae*, *Botourmiaviridae*, *Narnaviridae*, *Mitoviridae*, *Tymoviridae*, *Endornaviridae*, *Cystoviridae* and *Totiviridae* were created considering close amino acid viral sequences and ICTV reference sequences. For each dataset, a global alignment was created using the online tool MAFFT [62]. The alignment was visualized and trimmed with AliView [63]. The edited alignment was used as input in the program ModelTest to select the best evolutionary amino acid model according to the Akaike information criterion (AIC), and then a maximum likelihood phylogeny tree was inferred with 1000 bootstrap replicates. This last step was performed with the CIPRES Science Gateway [64]. The trees were visualized in the online tool Interactive Tree of Life (ITOL v6) [65]. Lastly, to search for conserved RNA-dependent RNA polymerase (RdRp) motifs within the putative viral proteins, we used the NCBI CDD, v3.20-59693 PSSM search tool with standard parameters [66].

### 2.4. Genetic Diversity of Species and Abundance of Viral Sequences

The assembled consolidated transcripts derived from SPAdes, rnaviralSPAdes and Trinity assemblies were analyzed using the Kaiju platform [67] with standard parameters (Supplementary Table S4). In addition, to produce the reference transcriptome, we performed a second contig assembly using the programs SPAdes (version 3.15.4 + galaxy1) [44], rnaviralSPAdes (version 3.15.4 + galaxy1) [45] and Trinity (version 2.9.1 + galaxy2) [46], with raw reads followed by TransDecoder (version 5.5.0 + galaxy2) [68] to identify high-confidence transcripts. Finally, the Salmon quant software (version 1.5.1 + galaxy0) [69] was executed using the identified transcripts and viral sequences to assess viral abundance. The host mitochondrial ribosomal protein S3 and nuclear beta-tubulin genes were selected as endogenous and standard genes for comparison with viral quantification.

### 2.5. Characterization of RNAi-Related Proteins in C. fimbriata

*Identification*: The proteins involved in the RNAi pathway, Dicer proteins 1 and 2 (DCL1 and DCL1), RNase III domain-containing protein (MRPL3), quelling deficient element genes (QDE), QDE-2-interacting protein (QIP), suppressor of ascus dominance (SAD) and suppressor of meiotic silencing (SMS) were identified based on annotation derived from *Neurospora crassa* [32]. The *N. crassa* proteins were aligned against the *Ceratocystis fimbriata* annotation (Cfim3.0 NCBI) using sequence similarity searches with the Blast software. The homologous proteins identified were evaluated for the presence of conserved domain characteristics of each class of proteins using the InterProScan 5.59–91.0 tool. *Dendrogram*: Homologous proteins from *N. crassa* and *C. fimbriata* were aligned by the ClustalW tool [70], and analyzed with the parameters of distance calculation, fractional dissimilarity and bootstrap value of 100. The location and name of the identified domains of each sequence were used to construct the domain plot of the dendrogram with the Archeopteryx 0.9914 tool [71]. *Abundance*: Transcripts corresponding to RNAi-related proteins in *C. fimbriata* were identified in their corresponding genome (Cfim3.0 NCBI) and used as a reference for quantification. The *C. fimbriata* transcriptome library (SRR16204154) was used for quantification, using the Salmon quant tool of the Galaxy platform with standard parameters.

## 3. Results

### 3.1. Transcriptome Assembly

The *C. cacaofunesta* (Cc) library presented 55,951,393 raw reads. After the quality filtering, 55,529,085 reads with Phred quality >20 were retained for further analysis. A total of 91.15% of the reads were aligned with the fungal reference genome. The reads that were not aligned with the reference genome were used in the transcriptome assembly. The assembly produced 1750 SPAdes transcripts, 965 rnaviralSPAdes transcripts and 480 Trinity transcripts. The consolidation and transcript extension step with CAP3 resulted in 1002 unambiguous sequences with N50 of 1932 and an average length of 892.9 nt. The *C. fimbriata* (Cf) library consisted of 42,480,313 raw reads. A total of 68.55% of the reads were aligned with the reference genome. The remaining reads were used in the assembly step, producing 7398 SPAdes transcripts, 10,709 rnaviralSPAdes transcripts and 64,961 Trinity transcripts. Transcript consolidation resulted in 52,493 nonredundant sequences with N50 of 729 and an average length of 593 nt (Supplementary Table S1).

### 3.2. Metagenomic Analysis

**Figure 1:**
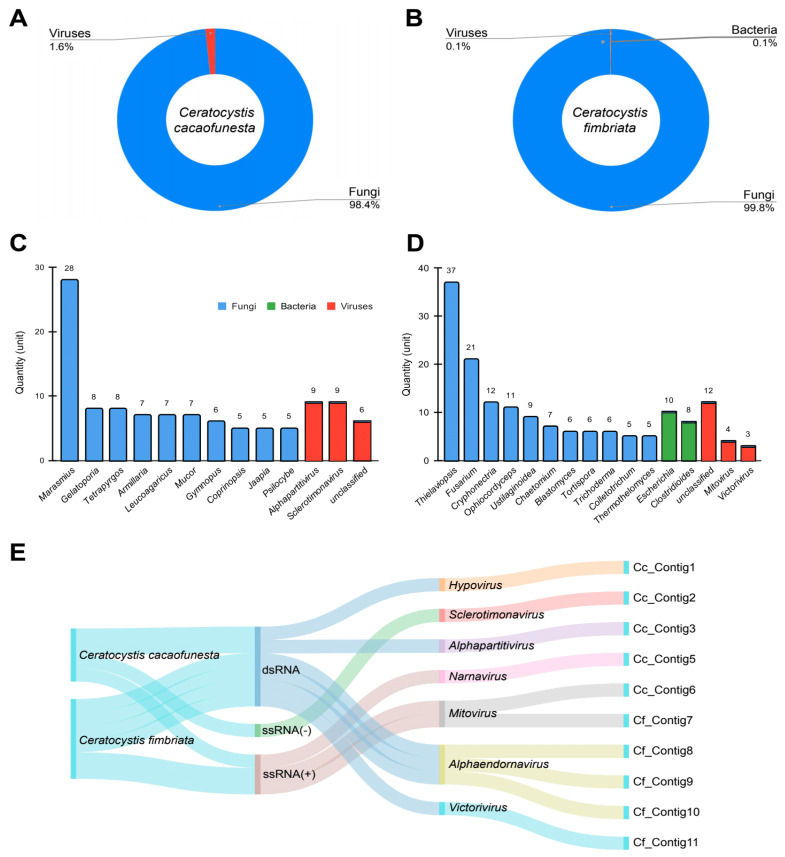
Metagenomic analysis of *Ceratocystis* samples. Diversity of microorganisms identified in *C. cacaofunesta* and *C. fimbriata* at the kingdom (**A**,**B**) and genus (**C**,**D**) level, respectively. (**E**) Sankey plot referring to the general diversity of transcripts derived from viruses identified in *Ceratocystis* samples classified by genome composition and viral family.

Transcriptomic data resulting from the consolidation of the data from the three different assemblers were used for initial screening using a metagenomics-based strategy with Kaiju. We observed transcripts derived mainly from fungi (Cc: 98.4%, Cf: 99.8%) and viruses (Cc: 1.6%, Cf: 0.1%) in *Ceratocystis* samples, with the exception of *C. fimbriata*, in which we also observed sequences derived from bacteria (0.1%) (Figure 1A,B). Regarding fungal diversity, in the sample derived from *C. cacaofunesta*, we observed the presence of transcripts assigned to species from different genera, such as *Marasmius*, *Gelatoporia* and *Armilaria*, while in *C. fimbriata*, we detected sequences related to *Thielaviopsis*, *Fusarium*, *Trichoderma* and *Colletotrichum*, among others (Figure 1C,D). The viral sequences detected included members of alphapartitivirus, sclerotimonavirus, an unclassified genus in *C. cacaofunesta* and mitovirus, victorivirus and an unclassified genus in *C. fimbriata* (Figure 1C,D). Sequences derived from bacteria were only identified in *C. fimbriata* and were restricted to species related to *Escherichia* and *Clostridioides* (Figure 1D).

### 3.3. Virome Characterization

We identified a considerable number of viral sequences in the metagenomicanalysis, so we further explored the presence of viruses in *Ceratocystis* samples. Therefore, we performed regular sequence similarity searches against NCBI nonredundant databases. For *C. cacaofunesta* and *C. fimbriata*, viral analysis identified the presence of double-stranded RNA viruses (dsRNA) and positive single-stranded RNA viruses (ssRNA+), while only in the case of *C. cacaofunesta*, we also identified elements relative to negative single-stranded RNA viruses (ssRNA−) (Figure 1E). At the genus level, members of the dsRNA genome included hypovirus, alphapartitivirus, alphaendornavirus and victorivirus, while ssRNA(+) was represented by narnavirus and mitovirus and, finally, ssRNA(−) by sclerotimonavirus (Figure 1E).

- *Hypoviridae*

**Figure 2:**
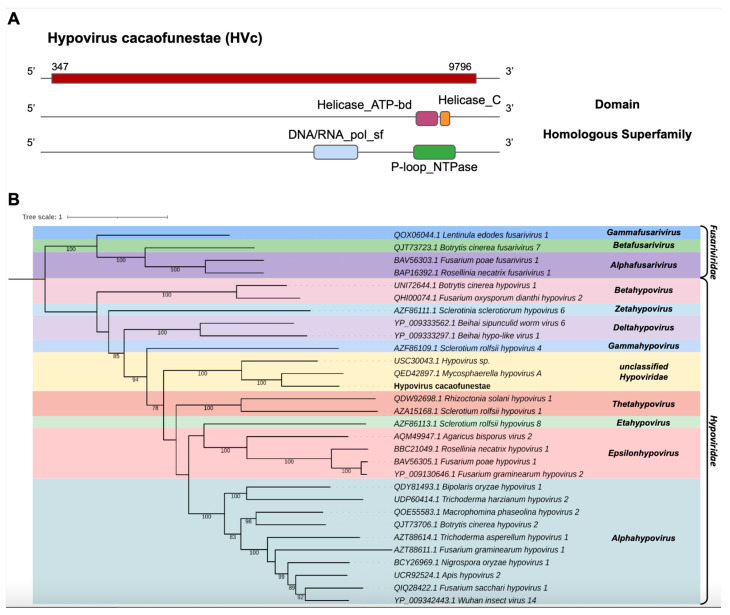
Characterization of the new *Hypovirus*. (**A**) Schematic representation of the novel hypovirus HVc with the ORF and domains represented. (**B**) Phylogenetic analysis using the ModelTest-NG according to the Akaike information criterion (AIC) indicated that the best evolutionary model was BLOSUM62 + F. Bootstrap values were established from 1000 replicates. Bootstrap values (%) lower than 70 are not shown.

The transcript assembled from the library of *C. cacaofunesta*, Cc_Contig1, presented 9920 nt and had similarity to viruses of the family *Hypoviridae* at the amino acid level. Structural annotation using NCBI ORFfinder indicated a large open reading frame (ORF) of 9450 nt surrounded by UTRs of 5′ and 3′ with 347 nt and 124 nt, respectively (Figure 2A). In Cc_Contig1, we detected the domains Helicase_ATP-bd (IPR014001) and Helicase_C (IPR001650) superimposed on the homologous superfamily P-loop_NTPase (IPR027417) and the DNA/ARN_pol_sf superfamily (IPR043502) (Figure 2A). According to the phylogenetic analysis, the putative virus was grouped with sequences from the genus *Hypovirus* (*Hypoviridae*), closely related to *Mycosphaerella hypovirus A* (MHV-A), with 100% bootstrap (Figure 2B). Indeed, analysis of conserved domains of the closely related MHV-A revealed the same domains identified in the Cc_Contig1, with the exception of the RNA-dir_pol_PSvirus domain (IPR007094), only identified in the MHV-A (Figure 2A and Supplementary Figure S1). This new virus was named Hypovirus cacaofunestae (HVc) to reflect the host origin. 

Of note, in the HVc viral contig, we also identified conserved domains (CDD) that presented a central catalytic domain of RdRp of RNA viruses belonging to the *Hypoviridae* family, ps-ssRNAv_Hypoviridae_RdRp (cd23170), which is a member of the ps-ssRNAv_RdRp-like superfamily (cl40470) located in the interval 2079–23038 and with an E-value of 1.53 × 10^−20^. This region represents conserved polymerase motifs called conserved polymerase motif A, located at position 2149–2163 TRFSADITAYDANTP; polymerase B motif, located at positions 2217–2233 and 2235–2240 GGTGQSSTSWDNHWGMR..AMIMIW; and conserved polymerase C motif, located at positions 2255–2258 and 2259–2269 NSVH..TGDDNIWGTD, sites that are found in seven (there are only six identified) viral species, namely, *Cryphonectria hypovirus* 3 (AAF13604), *Phomopsis longicolla hypovirus* (YP_009051683), *Valsa ceratosperma hypovirus* 1 (YP_005476604), *Sclerotinia sclerotiorum hypovirus* 1 (YP_004782527), *Cryphonectria hypovirus* 4 (YP_138519) and *Setosphaeria turcica hypovirus* 1 (AZT8861) (Supplementary Figure S2). In addition to the previously mentioned conserved motifs, this viral sequence presented a DEAD-like_helicase_N superfamily (smart00487) located in the interval 2722–2880 with an E-value of 3.82 × 10^−9^, which contains a DEAD box helicase motif DEFH, located at position 2818–2821.

- *Mymonaviridae*

**Figure 3:**
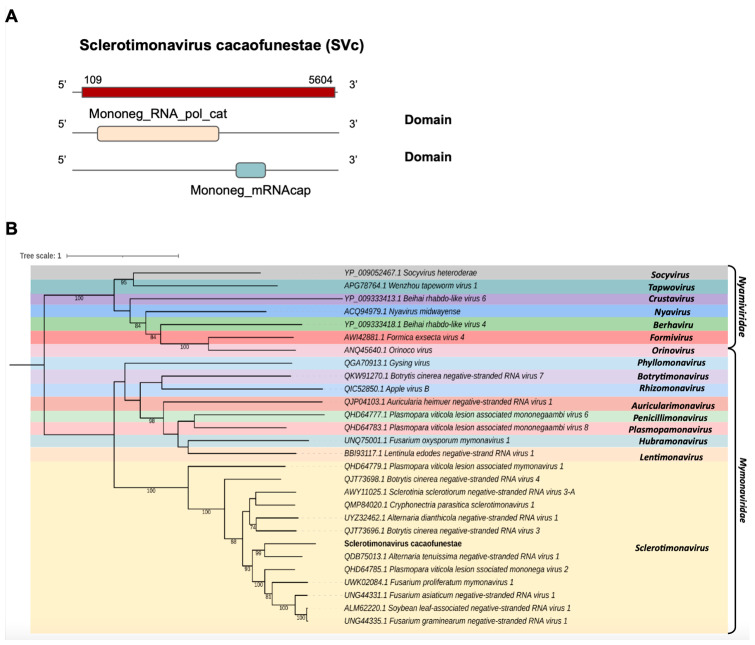
Conserved domains and phylogenetic analysis of sequences showing similarity to *Sclerotimonavirus*. (**A**) Schematic representation of the novel sclerotimonavirus SVc with the ORF and domains represented. (**B**) Phylogenetic analysis using the ModelTest-NG according to the Akaike information criterion (AIC) indicated that the best evolutionary model was BLOSUM62 + F. Bootstrap values were established with 1000 replicates. Bootstrap values (%) lower than 70 are not shown.

One transcript assembled from the *C. cacaofunesta* library, Cc_Contig2, with a size of 5.652 nt, showed similarity to viruses from the *Mymonaviridae* family at the amino acid level. Structural annotation indicated a large ORF of 5496 nt surrounded by 5′ and 3′ UTRs of 109 nt and 48 nt, respectively (Figure 3A). Conserved domains were identified in Cc_Contig2, such as Mononeg_RNA_pol_cat (IPR014023) and Mononeg_mRNAcap (IPR026890) (Figure 3A). Analysis of conserved domains of the closely related *Alternaria tenuissima negative-stranded RNA virus* 1 (AtNSRV1) revealed a very similar ORF length and the presence of the same domains identified in Cc_Contig2 (Figure 3A and Supplementary Figure S1). Phylogenetic analysis of the putative virus indicated a relationship with members of the genus *Sclerotimonavirus* (*Mymonaviridae*), closely related to *Alternaria tenuissima negative-stranded RNA virus* 1, with 99% bootstrap (Figure 3B). This new virus was named Sclerotimonavirus cacaofunestae (SVc).

- *Partitiviridae*

**Figure 4:**
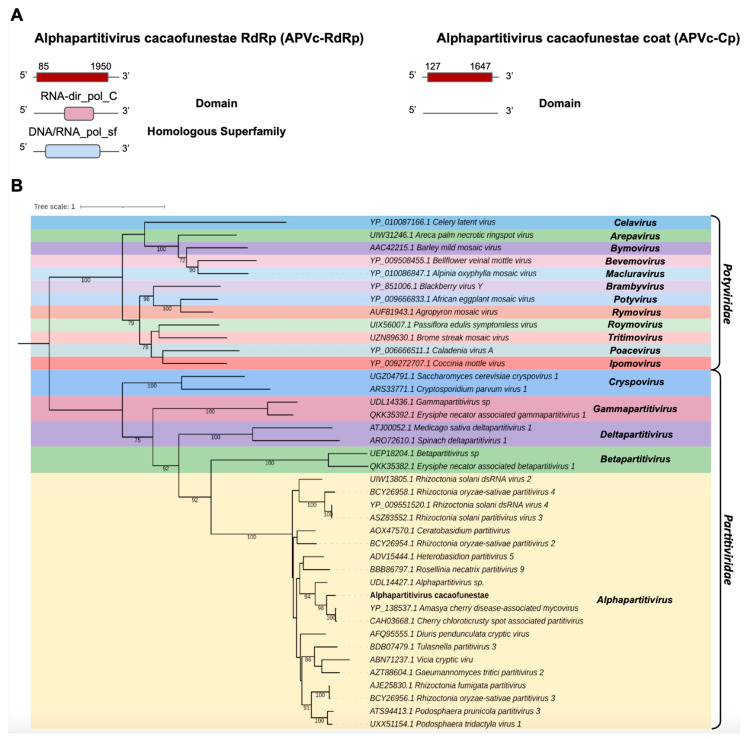
Characterization of the virus related to members of the *Partitiviridae* family. (**A**) Schematic representation of the novel partitivirus segments APVc-RdRp and APVc-Cp with the ORF and domains represented. (**B**) Phylogenetic analysis using the ModelTest-NG according to the Akaike information criterion (AIC) indicated that the best evolutionary model was VT + F. Bootstrap values were established with 1000 replicates. Bootstrap values (%) lower than 70 are not shown.

The transcript Cc_Contig3 assembled in the *C. cacaofunesta* library with a size of 1998 nt was closely related to viruses from the *Partitiviridae* family at the amino acid level. Structural annotation indicated a large ORF of 1866 nt surrounded by 5′ and 3′ UTRs of 85 nt and 48 nt, respectively. Another transcript from the *C. cacaofunesta* library, Cc_Contig4-Cp, with a size of 1818 nt, also showed similarity to viruses of the *Partitiviridae family*. This transcript was related to a coat protein whose structural annotation indicated an ORF of 1521 nt surrounded by 5′ and 3′ UTRs of 172 nt and 126 nt, respectively. Analysis of conserved domains in Cc_Contig3 revealed the presence of RNA-dir_pol_C (IPR001205) superimposed on the homologous superfamily DNA/RNA_pol_sf (IPR043502). On the other hand, Cc_Contig4-Cp did not have conserved domains (Figure 4A). Search for domains in the closest virus *Amasya cherry disease-associated mycovirus* (AcDAV) RdRp (currently *Cherry chlorotic rusty spot associated partitivirus*) revealed the presence of the same domains identified in Cc_Contig3 (Figure 4A and Supplementary Figure S1). The coat segment of AcDAV did not present domains, which was also true for Cc_contig4-Cp. According to the phylogenetic analysis, the putative virus is related to sequences from the genus *Alphapartitivirus* (*Partitiviridae*), closely related to the *Amasya cherry disease-associated mycovirus* (AcDAV) grouping with 98% bootstrap (Figure 4B). Therefore, the virus was named Alphapartitivirus cacaofunestae (APVc-RdRp).

- *Narnaviridae and Mitoviridae*

**Figure 5:**
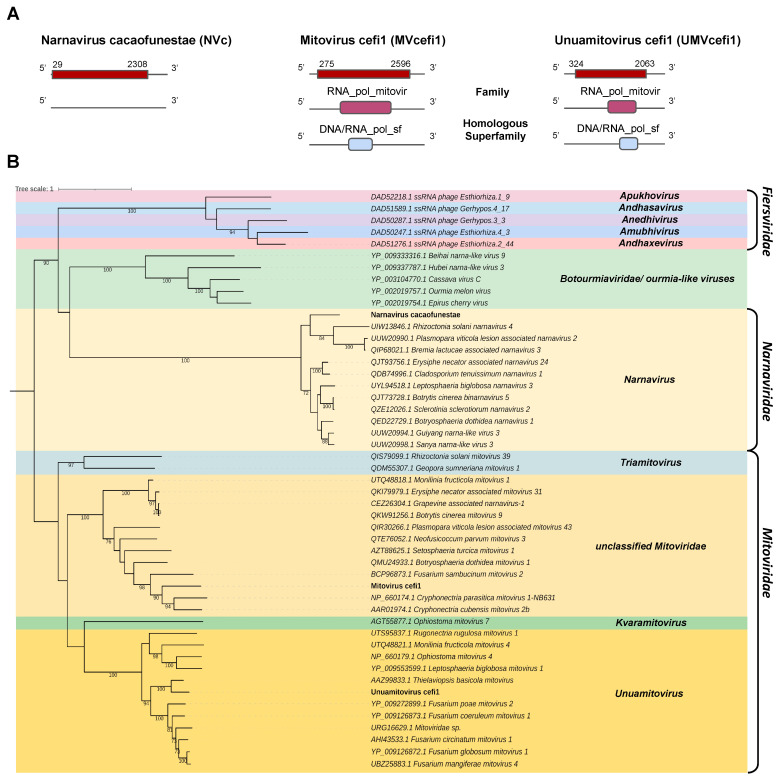
Phylogenetic analysis of sequences showing similarity to *Narnavirus*, *Mitovirus* and *Unuamitovirus*. (**A**) Schematic representation of the novel narnavirus NVc and mitoviruses MVcefi1 and UMVcefi1 with the ORF and domains represented. (**B**) Phylogenetic analysis using the ModelTest-NG according to the Akaike information criterion (AIC) indicated that the best evolutionary model was VT + F. Bootstrap values were established with 1000 replicates. Bootstrap values (%) lower than 70 are not shown.

Two transcripts assembled from the *C. fimbriata* library and the *C. cacaofunesta* library showed sequence similarity to members of the families *Narnaviridae* and *Mitoviridae*. The contig belonging to the *C. cacaofunesta* library, Cc_Contig5, with a size of 2409 nt, showed similarity to viruses of the *Narnaviridae* at the amino acid level. Structural annotation of Cc_Contig5 indicated an ORF of 2381 nt surrounded by UTRs of 3′ and 5′ with 28 nt and 102 nt, respectively. The second contig assembled from the *C. fimbriata* library, Cf_Contig6, presented a length of 3268 nt and showed a large ORF of 2322 nt surrounded by UTRs of 3′ and 5′ with 275 nt and 672 nt, respectively. The other contig assembled from the *C. fimbriata* library, Cf_Contig7, presented 2395 nt, and the structural annotation indicated an ORF of 1740 nt surrounded by UTRs of 5′ and 3′ of 324 nt and 332 nt, respectively. These two transcripts showed similarity to viruses of the *Mitoviridae* at the amino acid level. Of note, all three transcripts only presented large ORFs in genetic code 4 (Mold, Protozoan Mitochondrial and Coelenterate Mitochondrial and the Mycoplasma/Spiroplasma) consistent with the replication strategy of narnaviruses and mitoviruses. Cc_Contig5 did not present conserved domains. However, for Cf_Contig6 and Cf_Contig7 sequences, we observed the presence of the RNA_pol_mitovir family (IPR008686), which overlapped with the homologous DNA/RNA_pol_sf superfamily (IPR043502) (Figure 5A).

Analysis of conserved domains of the closely related *Botrytis cinerea binarnavirus* 5 (BcBNV5), *Cryphonectria parasitica mitovirus* 1-NB63 1(CpMV1) and *Thielaviopsis basicola mitovirus* (TbMV) revealed the same domain profile observed in the transcripts Cc_Contig5, Cc_Contig6 and Cc_Contig7, respectively. The exception was the RT_dom domain (IPR000477), only identified in *Thielaviopsis basicola mitovirus* (TbMV) (Figure 5A and Supplementary Figure S1).

According to the phylogenetic analysis, the putative virus represented by the transcript Cc_Contig5 was grouped with members of the genus *Narnavirus* (*Narnaviridae*) within the clade containing BcBNV5 (Figure 5B). Cf_Contig6 formed a clade containing members of *Mitoviridae* but without genus designation. Finally, Cf_Contig7 was grouped with sequences from the genus *Unuamitovirus* (*Mitoviridae*), with 100% bootstrap. (Figure 5B). These new viruses were named Narnavirus cacaofunestae (NVc), Mitovirus cefi1 (MVcefi1) and Unuamitovirus cefi1 (UMVcefi1), respectively.

Further analysis regarding conserved motifs revealed that the NVc viral transcript presented a central conserved RNA-dependent RNA polymerase (RdRp) catalytic domain ps-ssRNAv_RdRp-like superfamily (cl40470), located in the interval 527–736 with an E-value of 2.35 × 10^−7^. This region contained two conserved polymerase motifs called conserved polymerase A motifs, located at positions 612 and 631–644 D..FSSTDYQEATDAMQ, and conserved polymerase B motifs, located at positions 698–724 and 726–729 VLMGDPLTKPVLHLVNI..VRRI. Both motifs were found in the ps-ssRNAv_Botourmiaviridae_RdRp domain (cd23183) belonging to the *Botourmiaviridae* family, order *Ourlivirales* (Supplementary Figure S2).

- *Endornaviridae*

**Figure 6:**
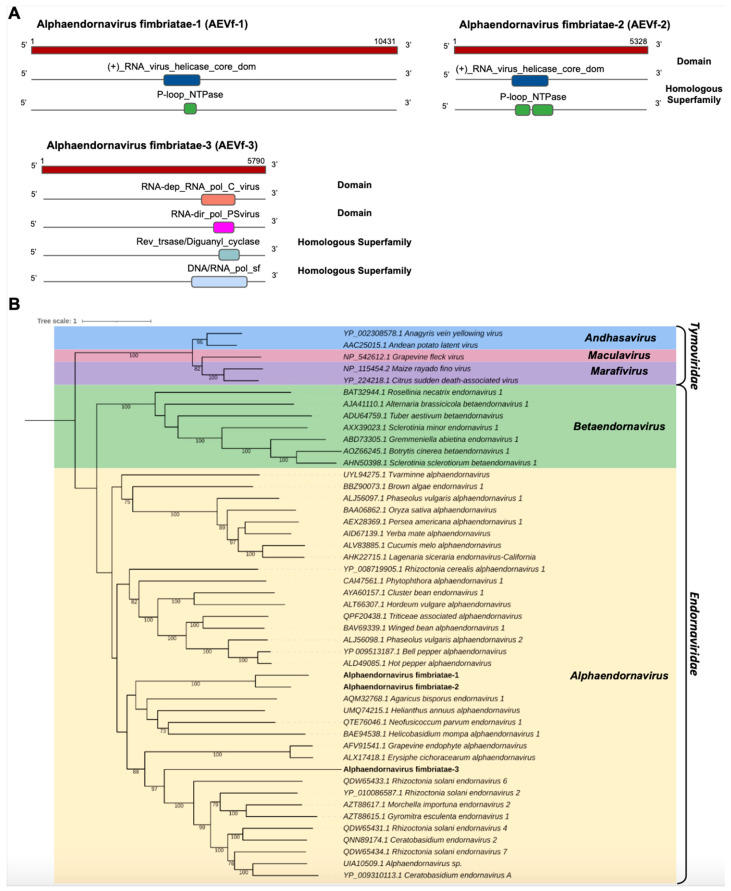
Conserved domains and phylogenetic analysis of sequences showing similarity to *Alphaendornavirus*. (**A**) Schematic representation of the novel alphaendornaviruses AEVf-1, AEVf-2 and AEVf-3 with the ORF and domains represented. (**B**) Phylogenetic analysis using the ModelTest-NG according to the Akaike information criterion (AIC) indicated that the best evolutionary model was BLOSUM62 + F. Bootstrap values were established with 1000 replicates. Bootstrap values (%) lower than 70 are not shown.

We identified three transcripts in the *C. fimbriata* library showing sequence similarity to members of the *Endornaviridae* family. Cf_Contig8, with a length of 10,432 nt, presented an ORF of 10,431 nt surrounded by UTRs of 5′ and 3′ with 1 nt and 834 nt, respectively. For Cf_Contig9, we only reconstituted a fragment with an expected length of 5328, likely representing a fragment of the large polyprotein gene. Similarly, Cf_Contig10 of 5790 nt denoted a partial ORF. All of them showed similarity to *Endornaviruses* at the amino acid level. Regarding the presence of conserved domains, Cf_Contig8 and Cf_Contig9 contained the RNA_virus_helicase_core_dom domain (IPR027351) superimposed on the homologous superfamily P-loop_NTPase (IPR027417), while Cf_Contig10 showed the RNA-dep_RNA_pol_C_virus (IPR001788) domain with integration of the conserved RNA-dir_pol_PSvirus (IPR007094) domains found to overlap with the homologous superfamily Rev_trsase/Diguanyl_cyclase (IPR043128) integrated with DNA/RNA_pol_sf (IPR043502) (Figure 6A). 

Analysis of conserved domains of the *Tvarminne alphaendornavirus*, the closest viruses to Cf_Contig8, revealed the presence of shared domains, with the exception of the RNA-dep_RNA_pol_C_virus (IPR001788), RNA-dir_pol_PSvirus (IPR007094) and DNA/RNA_pol_sf (IPR043502) domains. The analysis of conserved domains of *Alphaendornavirus* sp., the closest virus to Cf_Contig9, revealed that they share two domains, with the exception of Mycovirus_RNAse (IPR046747), RNA-dep_RNA_pol_C_virus (IPR001788), RNA-dir_pol_PSvirus (IPR007094) and DNA/RNA_pol_sf (IPR043502). The analysis of conserved domains of *Morchella importuna endornavirus* 2 (MiEV2), the closest virus to Cf_Contig10, revealed that they also share two domains, with the exception of (+)_RNA_virus_helicase_core_dom (IPR027351) and P-loop_NTPase (IPR027417) only identified in MiEV2 and RNA-dir_pol_PSvirus (IPR007094) and Rev_trsase/Diguanyl_cyclase (IPR043128) only identified in Cf_Contig10 (Figure 6A and Supplementary Figure S1). 

The phylogeny of the putative viruses revealed that Cf_Contig8 and Cf_Contig9 were grouped together and, like Cf_Contig10, were closely related to members of the *Alphaendornavirus* genus (*Endornaviridae*) (Figure 6B). These new viruses were named Alphaendornavirus fimbriatae-1 (AEVf-1), Alphaendornavirus fimbriatae-2 (AEVf-2) and Alphaendornavirus fimbriatae-3 (AEVf-3), respectively.

AEVf-3 also presented a conserved central catalytic domain of the RNA-dependent RNA polymerase (RdRp) ps-ssRNAv_RdRp-like super family (cl40470), located in the interval 1491–1736 with an E-value of 1.52 × 10^−118^. This region represents conserved polymerase motifs called conserved polymerase A motif, located at position 2613–2627 LFVEDDLEKQDRQTD; conserved polymerase B motif, located at position 1673–1696 RLTGQATTALGNVITNLLVHSRLV; and conserved polymerase C motif, located at position 1704–1718 VIMMCLGDDNLMVCR. All motifs are found in the Endornaviridae_RdRp (cd23255) domain, belonging to the *Endornaviridae* family (Supplementary Figure S4).

- *Totiviridae*

**Figure 7:**
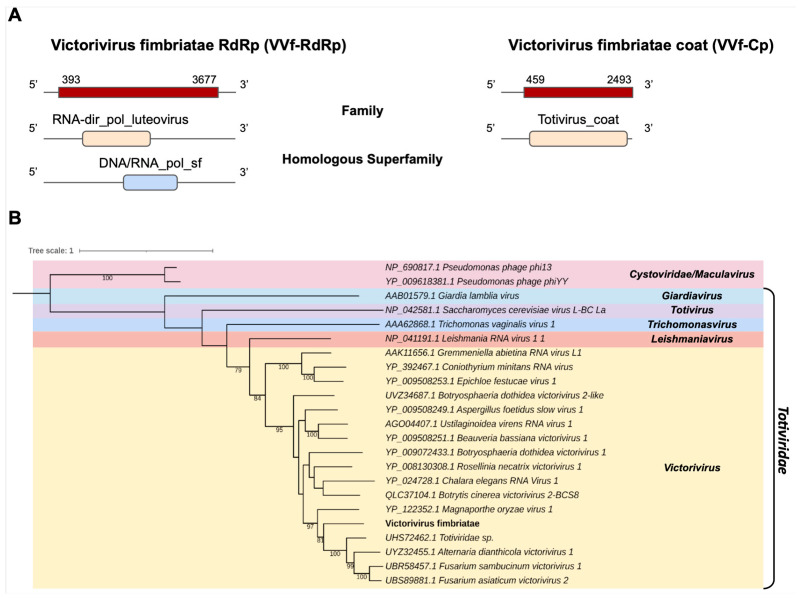
Conserved domains and phylogenetic analysis of sequence showing similarity to *Victorivirus*. (**A**) Schematic representation of the novel victorivirus segments VVf-RdRp and VVf-Cp with the ORF and domains represented. (**B**) Phylogenetic analysis using the ModelTest-NG according to the Akaike information criterion (AIC) indicated that the best evolutionary model was BLOSUM62 + F. Bootstrap values were established with 1000 replicates. *Cystovirus* was used as an outgroup to root the tree. Bootstrap values (%) lower than 70 are not shown.

One of the transcripts assembled in the *C. fimbriata* library, Cf_Contig11, with a length of 3995 nt was related to viruses from the *Totiviridae* family at the amino acid level. Cf_Contig11 contained an ORF of 3285 nt surrounded by 5′ and 3′ UTRs with 393 nt and 318 nt, respectively. We observed that the presence of the conserved domain RNA-dir_pol_luteovirus family (IPR001795) overlapped with the homologue superfamily DNA/RNA_pol_sf (IPR043502). Another transcript in the *C. fimbriata* library, Cf_Contig12, with a size of 2493 nt also showed similarity to viruses of the *Totiviridae* at the amino acid level, with an ORF of 2034 nt surrounded by 5′ and 3′ UTRs with 459 nt and 1 nt, respectively, and presenting the conserved domain for the Totivirus_coat family (IPR008871) (Figure 7A). The analysis of conserved domains of the closely related *Totiviridae* sp. sequence revealed the presence of the same domains observed for Cf_Contig11 (Figure 7A and Supplementary Figure S1). 

According to the phylogenetic analysis, the putative virus was grouped with sequences from the genus *Victorivirus* (*Totiviridae*), closely related to *Totiviridae* sp. (Figure 7B). This new virus was named Victorivirus fimbriatae (VVf).

### 3.4. Abundance of Viral Sequences

**Figure 8:**
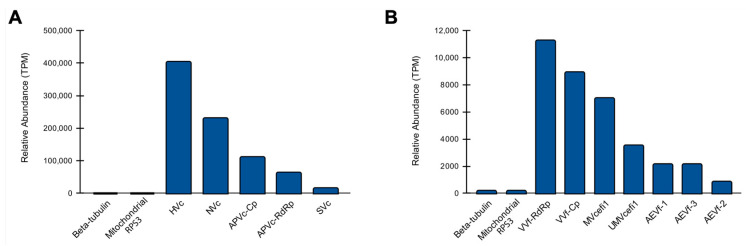
Abundance of viral sequences in Ceratocystis samples. Relative abundance of new viruses identified in *C. cacaofunesta* (**A**) and *C. fimbriata* (**B**). For both organisms, we included the quantification of the constitutive genes beta-tubulin and mitochondrial RPS3.

Unexpectedly, we observed a complex diversity of viruses infecting the fungal species. Therefore, we decided to investigate the abundance of these viral species in comparison with endogenous nuclear and mitochondrial genes. Regarding the putative viruses identified in *C. cacaofunesta*, HVc showed the highest abundance with 404.689 TPM, while SVc presented the lowest with 15.258 TPM (Figure 8A). Of note, the constitutive genes beta-tubulin and mitochondrial RPS3 accounted for 0.081 and 0.253 TPM, respectively. Our results suggest that the transcriptional activity of the *C. cacaofunesta*-infecting viruses is ~180 to ~5000 times higher than the constitutive nuclear gene (Figure 8A). In the case of viruses identified in *C. fimbriata*, VVf had the greatest abundance with 11.289 TPM, while AEVf-2 showed the lowest with 0.846 TPM (Figure 8B). Unlike what was observed for *C. cacaofunesta*, *C. fimbriata*-infecting viruses showed a transcriptional activity closer to that detected for the marker genes beta-tubulin and mitochondrial RPS3, with less than a 10-fold difference (Figure 8B).

### 3.5. Characterization of RNA-Interference-Related Genes in Ceratocystis Fimbriata

Other studies have shown that fungi can contain RNA interference (RNAi) machinery with important roles in genome stability and antiviral response [32]. Therefore, we hypothesized that RNAi could be involved in the management of multiple viral infections by fungi. Thus, we investigated whether their genomes contained the RNAi core genes and whether they were transcriptionally active. Since the genome of *C. cacaofunesta* is not annotated, we performed characterization of RNAi genes in *C. fimbriata*. Using proteins identified and validated experimentally in the model fungus *Neurospora crassa*, we searched for RNAi-related genes including the RNA-dependent RNA polymerases QDE-1 and SAD-1, Argonaute-2 orthologs, QDE-2 and SMS2 and Dicer, among others (Supplementary Table S5 and Figure S5A). The RNAi-related proteins identified had similar lengths and presence of conserved domains, including all canonical elements expected for Argonaute and Dicer genes (Supplementary Table S5 and Supplementary Figure S5A). Quantification of RNAi-related genes detected transcriptional activity for all of the genes assessed, with Argonaute orthologues showing higher abundance and SAD and SMS2 presenting the lowest number of transcripts detected.

## 4. Discussion

*Ceratocystis* in general accommodates many important pathogens of agricultural crops and woody plants. *C. fimbriata* was the first to be described and can be found in plantations mainly of *Ipomoea batatas* [11,72,73,74], *Mangifera indica* [75,76], *Eucalyptus* sp. [75,77,78], *Ficus carica* [75,79], *Punica granatum* [80,81], *Colocasia esculenta* [80,82,83], *Theobroma cacao* [11], *Gmelina arborea* [84,85], *Syngonium* sp. [86,87] and *Ilex paraguariensis* [88,89]. In contrast, *C. cacaofunesta* has only been described in *Theobroma cacao* [90] and in species from the genus *Herrania* sp. [91].

Metatranscriptomic analyses indicated the presence of several microorganisms in the libraries, including elements derived from bacteria and viruses. This diversity of microorganisms may be related to the culture medium used for fungal growth, which was enriched with cocoa xylem of *C. cacaofunesta* [10] and was extracted from an isolate preserved in a culture collection and subjected to genome sequencing of *C. fimbriata* [39,40]. Nevertheless, the possible presence of these species does not interfere with the result, since the viral sequences assembled in our study are from families described as infecting fungi, not bacteria. Furthermore, their abundance was hundreds of times lower than that of *C. cacaofunesta* and *C. fimbriata*, inconsistent with the abundance of viruses in the sample.

*Hypoviridae* is a family of capsidless viruses that present a positive-sense ssRNA genome of 9.1 to 12.7 kb encoding one or two ORFs [92,93]. These characteristics are similar to those found in our research. In addition, when comparing the sequence called HVc with *Mycosphaerella hypovirus* A, we observed similarities in terms of the conserved domains found, demonstrating the probable membership of the new virus in the *Hypoviridae* family. It is worth mentioning that other studies have identified *Hypovirus* in other fungal species, such as the brown rot fungus *Monilinia fructicola* reported by De Miccolis Angelini and collaborators (2022) [94], who named them *Monilinia fructicola hypovirus* 1 and *Monilinia fructicola hypovirus* 2, or MfrcHV1 and MfrcHV2, respectively. In MfrcHV1, the polyprotein had three conserved domains (an RdRp, a putative peptidase PPPDE and a C-terminal helicase), while MfrcHV2 had two conserved domains in the polyproteins (the superfamilies 1 and 2 of type 1 ATP binding and C-terminal helicases), presenting similarity with the conserved domains present in the viral contigs described in our study.

Other researchers who have reported the identification and characterization of hypoviruses are Velasco and collaborators (2018) [95], studying *Entoleuca* sp.; Li and collaborators (2020) [96], studying *Bipolaris oryzae;* Wang and collaborators (2022) [97], who studied *Fusarium graminearum hypovirus* 1 (FgHV1) and demonstrated that it has proteins with characteristics such as suppression of host RNA silencing; and Aulia and collaborators (2021) [98], who studied the fungus *Cryphonectria parasitica* and showed that it facilitates infection by inducing hypovirulence. The viral contig HVc was the first hypovirus detected in *Ceratocystis cacaofunesta.*

The viruses from the *Mymonaviridae* produce filamentous and enveloped virions containing a single negative-sense linear RNA molecule of about 10 kb. Mimonaviruses usually infect filamentous fungi, and one of the negative-strand RNAs of one virus, *Sclerotinia sclerotiorum* virus 1, induces hypovirulence in the fungal host [99]. Our phylogenetic analysis suggests a new virus genome belonging to this family, SVc. Furthermore, a previous study by Li and collaborators (2022) [100] characterized a new ssRNA(−) mycovirus isolated from the fungus *Auricularia heimuer* CCMJ1222, while another study, by Hao, Wu & Li (2018) [101], described an isolate of the phytopathogenic fungus *Botrytis cinerea.* The viral contig SVc was the first sclerotimonavirus detected in *Ceratocystis cacaofunesta.*

According to Vainio and collaborators (2018) [102], *Partitiviridae* is a family of small, isometric, nonenveloped viruses with bisegmented double-stranded (ds) RNA genomes of 3–4.8 kbp. The two genome segments are individually encapsulated and have a single large ORF or two ORFs, as in the current study where an ORF was presented, identifying and characterizing a new virus belonging to the *Partitiviridae* family, which presented a fragment of RdRp and a coat protein segment, which were named APVc-RdRp and APVc-CP, respectively, due to their phylogenetic proximity to alphapartitiviruses. Furthermore, Jiang and collaborators (2021) [103] studied the fungus *Aspergillus nidulans*, and Moriyama and collaborators (2021) [104] analyzed the phytopathogen *Rosellinia necatrix*, the causative agent of root rot, also identifying viral sequences related to this family. Finally, Deng & Boland (2007) [105] reported viruses belonging to the genus *Partiviridae* isolated from *Ceratocystis resinifera*, *called* named *Ceratocystis partitivirus* 1 CPV1. This virus has the same characteristics as the virus present in *Ceratocystis polonica.* The viral contigs APVc-RdRp and APVc-CP represent the first alphapartitivirus detected in *Ceratocystis cacaofunesta.*

In addition, a new viral contig called NVc belonging to *Narnaviridae* was identified. The contig showed similarity to individual RNA virus genomes ranging from 2.3 to 3.6 kb encoding only a single polypeptide having an RdRp domain. This feature was found in the study by Botella and collaborators (2022) [106], who described narnaviruses in the fungus *Phytophthora palustris*, viruses that are related to the traditional positive-coding monopartite, included in the genus *Narnavirus*. Also of note, Kinsella and collaborators (2022) [107] identified narnaviruses in the hosts *R. oryzae* and *A. lentulus*, which are pathogenic fungi, and Zou et al. (2021) [108] analyzed the fungus *Botryosphaeria dothidea*, which is the causal agent of the disease pear ring rot. The viral contig NVc is the first narnavirus detected in *Ceratocystis cacaofunesta.*

Two new viral contigs, named UMVcefi1 and MVcefi1, belonging to the *Mitoviridae* were characterized in our study. Members of this family are positive-stranded ssRNA viruses of 2.5 to 2.9 kb. Generally, the genome has a single ORF encoding an RdRp. Previous studies have also identified viral contigs in different fungal phytopathogens, such as Wang and collaborators (2021 A-B) [109,110] and de Rezende and collaborators (2021) [111]. Shafik and collaborators (2021) [112] identified a new mycovirus, named *Melanconiella theae mitovirus* 1 (MtMV1), a virus that infects the phytopathogenic MtMV1 and shares most of the characteristics of the members of the *Mitoviridae* family. The UMVcefi1 and MVcefi1 sequences represents the first mitoviruses detected in *Ceratocystis fimbriata*.

AEVf-1, AEVf-2 and AEVf-3 are similar to *Endornaviridae*, which has a linear dsRNA genome of about 14 kb to 17.6 kb. This genome encodes an ORF potentially divided into several polypeptides. Here, two major ORF-associated domains were found: *Tymovirus*, RdRp, and (+) RNA virus helicase core dom, which have been identified in several virus types [113] including a variety of plant virus families [114]. In addition, Luo and collaborators (2022) [115], analyzing *S. sclerotiorum*, identified a new *endornavirus* called *Scelrotinia sclerotiorum endornavirus* 11 (SsEV11), suggesting that there is remarkable phylogenetic diversity in the *Endornaviridae* family and corroborating previous findings that viruses of this family can be found in fungi. In this respect, Cao and collaborators (2022) [116] studied *Ceratobasidium* infected with three endornaviruses with significantly upregulated micro-RNA-like RNAs (Cer-milRNAs) and identified transcription factors, suggesting an effect of viral infection on the control of regulatory networks and growth and development of the fungus. Moreover, Marais (2021) [117] investigated *Neofusicoccum parvum* and found the presence of co-infecting viruses from *Totiviridae*, *Victorivirus*, *Endornaviridae*, *Mitoviridae* and *Narnaviridae*. The viral contigs AEVf-1, AEVf-2 and AEVf-3 are the first alphaendornaviruses belonging to the *Endornaviridae* family detected in *Ceratocystis fimbriata.*

Finally, the *Totiviridae* has the main feature of being a nonenveloped icosahedral virion composed of a single coat protein (Cp), with a diameter of about 40 nm and a linear dsRNA genome of 4.6 to 6.7 kb. In addition, it has two overlapping ORFs, gag and pol, respectively encoding Cp and RdRp. In the viral contig assembled in our study (VVf), we identified a conserved domain of RdRp *Luteovirus*, which is an essential protein encoded in the genomes of all RNA containing viruses with no DNA stage [118,119]. The viral contigs VVf-RdRp and VVf-Cp were the first representants of a *Victorivirus* detected in *Ceratocystis fimbriata.*

Many fungal species have been found able to counteract multiple viral infections relying on the RNA interference (RNAi) mechanisms with antiviral activity, such as described for *Cryphonectria parasitica* [29]. When the host’s RNAi mechanism targets the viral genome, an overlapping population of virus-derived vsiRNA can be produced. In *C. parasitica*, the dicer-like (dcl-2) and argonaute-like (agl-2) genes are necessary for antiviral silencing [27,120]. Shinji Honda (2020) [121] demonstrated that in the fungus *Neurospora crassa*, viral infection positively regulates the transcription of the various components of RNAi, where the Dicer (DCL-1 and DCL-2) and Argonaute (QDE-2) proteins participate in the suppression of viral replication. Mekhala Maiti (2007) [122] reported that the Argonaute QDE-2 homologue was necessary for the generation of single-stranded siRNA and gene silencing in vivo.

Although various RNAi pathways have been lost in several fungal species and lineages, most species possess this ancient mechanism [27,36]. However, there are several filamentous fungi that seem to lack the necessary components for RNA silencing to occur [27], which means that by lacking this machinery to defend against mycoviruses, these viruses produce different hypovirulence factors in their hosts. Mycovirus-mediated hypovirulence is an excellent example of multitrophic interactions, making these viruses promising for the biological control of fungal diseases [26,123].

In our study, the highest abundance of RNAi occurred in two Argonaute genes, QDE2 and SMS2, with a value of around 300 TPM, as previously indicated by Lee and collaborators (2009) [124] in their study of QDE genes induced by DNA damage. On the other hand, Li and collaborators. (2022) [100], studying *Fusarium oxysporum* f. sp. *cubense*, noted that by knocking out QDE2, the fungus showed reduced virulence, suggesting its involvement in pathogenesis. Another noteworthy fact is that the biogenesis of milR4 requires MRPL3 [32], which may explain its presence in the data analyzed. We can conclude that the genes have all the components of the RNAi pathway and are transcriptionally active, suggesting that the pathway may help the fungus combat viral infection.

Overall, of the *Ceratocystis* species reported to date, two stand out: *Ceratocystis fimbriata*, which has been reported to infect nearly 100 host species, including many woody plants [125,126], and *Ceratocystis cacaofunesta*, which has been reported in *Theobroma cacao* and in the genus *Herrania* [90,125,126]. Therefore, our study reveals new insights into the mycovirome diversity of *Ceratocystis*, an important pathogen that causes irreversible damage to the vascular system of cocoa plants, reducing yield and, in severe cases, killing the plant.

## Data Availability

The datasets generated and analyzed during the current study are available in the NCBI GenBank Third Party Annotation database under accession numbers BK062942-BK062946 and BK063053-BK063059.

## Supplementary Materials

The following supporting information can be downloaded at: https://www.mdpi.com/article/10.3390/pathogens12020287/s1, Table S1: Overview of RNA deep sequencing libraries used in the work; Table S2: Best hits at nucleotide and amino acid levels for viral transcripts assembled in our work; Table S3: Viral genomes used in phylogenetic analyses; Table S4: Metagenomic analysis of RNA sequencing libraries using Kaiju software; Table S5: RNAi-related genes; Figure S1: Genomic characteristics of viral genomes closely related to the viruses identified in our work; Figure S2: Conserved motifs and catalytic site in the Hypovirus cacaofunestae viral sequence; Figure S3: Conserved motifs and catalytic site in the Narnavirus cacaofunestae viral sequence; Figure S4: Conserved motifs and catalytic site in the Alphaendornavirus fimbriatae-3 viral sequence; Figure S5: Characterization of RNAi-related genes in *Ceratocystis fimbriata*.
