# Supplementary material for: Uncovering a Complex Virome Associated with the Cacao Pathogens Ceratocystis cacaofunesta and Ceratocystis fimbriata
Source: Pathogens. 2023 Feb 9;12(2):287. doi: 10.3390/pathogens12020287 (PMC9967352; doi:10.3390/pathogens12020287)
Supplement: Supplementary file 1 [file pathogens-12-00287-s001.zip › supplementary_material.pdf]

Uncovering a Complex Virome Associated with the Cacao Pathogens *Ceratocystis cacaofunesta* and *Ceratocystis fimbriata*

Roy Bogardid Ardón Espinal [1,†], Sabrina Ferreira de Santana [1,2,†], Vinícius Castro Santos [3], Gabriela Nicolle Ramos Lizardo [1], Raner José Santana Silva [1], Ronan Xavier Corrêa [1], Leandro Lopes Loguercio [1], Aristóteles Góes-Neto [3], Carlos Priminho Pirovani [1], Paula Luize Camargos Fonseca [1] and Eric Roberto Guimarães Rocha Aguiar [1,*]

[1] Center for Biotechnology and Genetics, Universidade Estadual de Santa Cruz, Ilhéus 45662-900, BA, Brazil
[2] Department of Microbiology, Universidade Federal da Bahia, Salvador 40170-115, BA, Brazil
[3] Department of Biochemistry and Immunology, Universidade Federal de Minas Gerais, Belo Horizonte 31270-901, MG, Brazil
* Correspondence: ericgdp@gmail.com; Tel.: +55-31-99202-0015
† These authors contributed equally to this work.

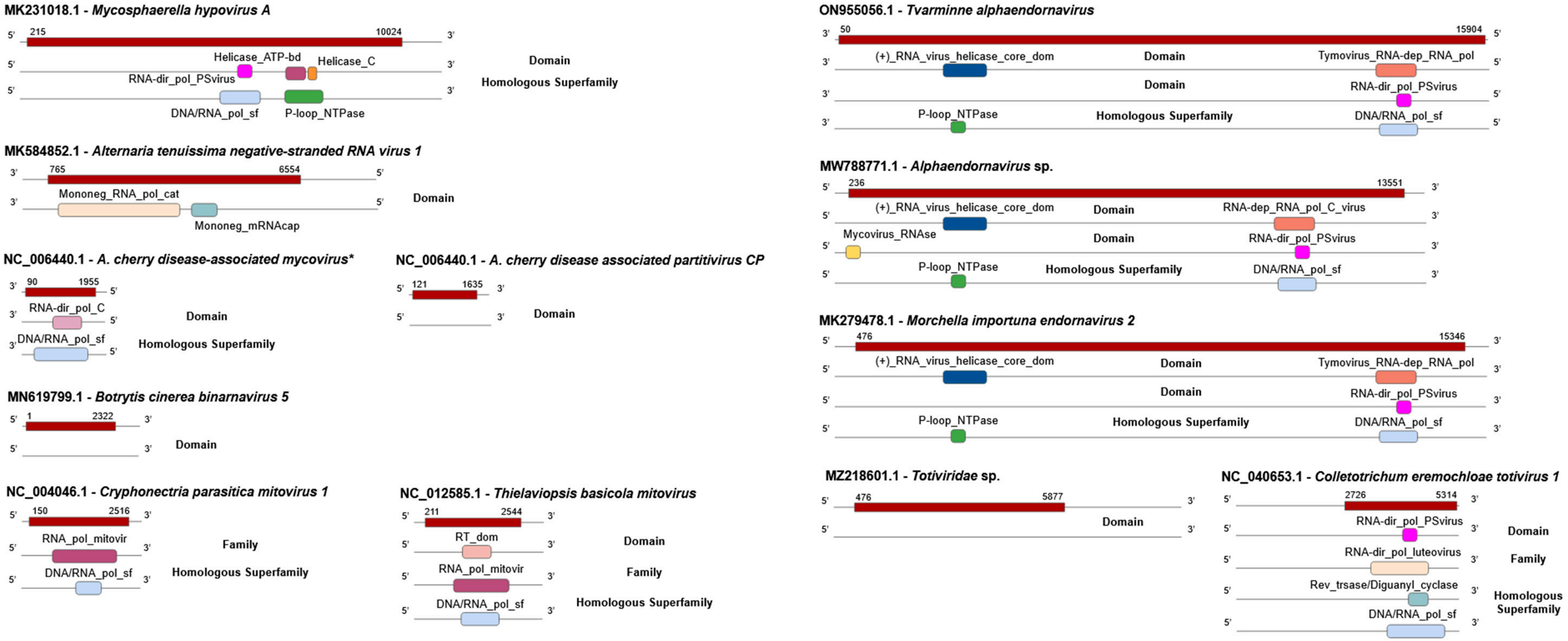

* *Amasya cherry disease-associated mycovirus* is a below-species classification of *Cherry chlorotic rusty spot associated partitivirus*

Figure S1. Conserved structures of the hit organisms for e-value of the studied sequences based on BlastX similarity analysis. The ORFfinder program was used to identify the largest ORF and InterProScan program was used to identify conserved structures.

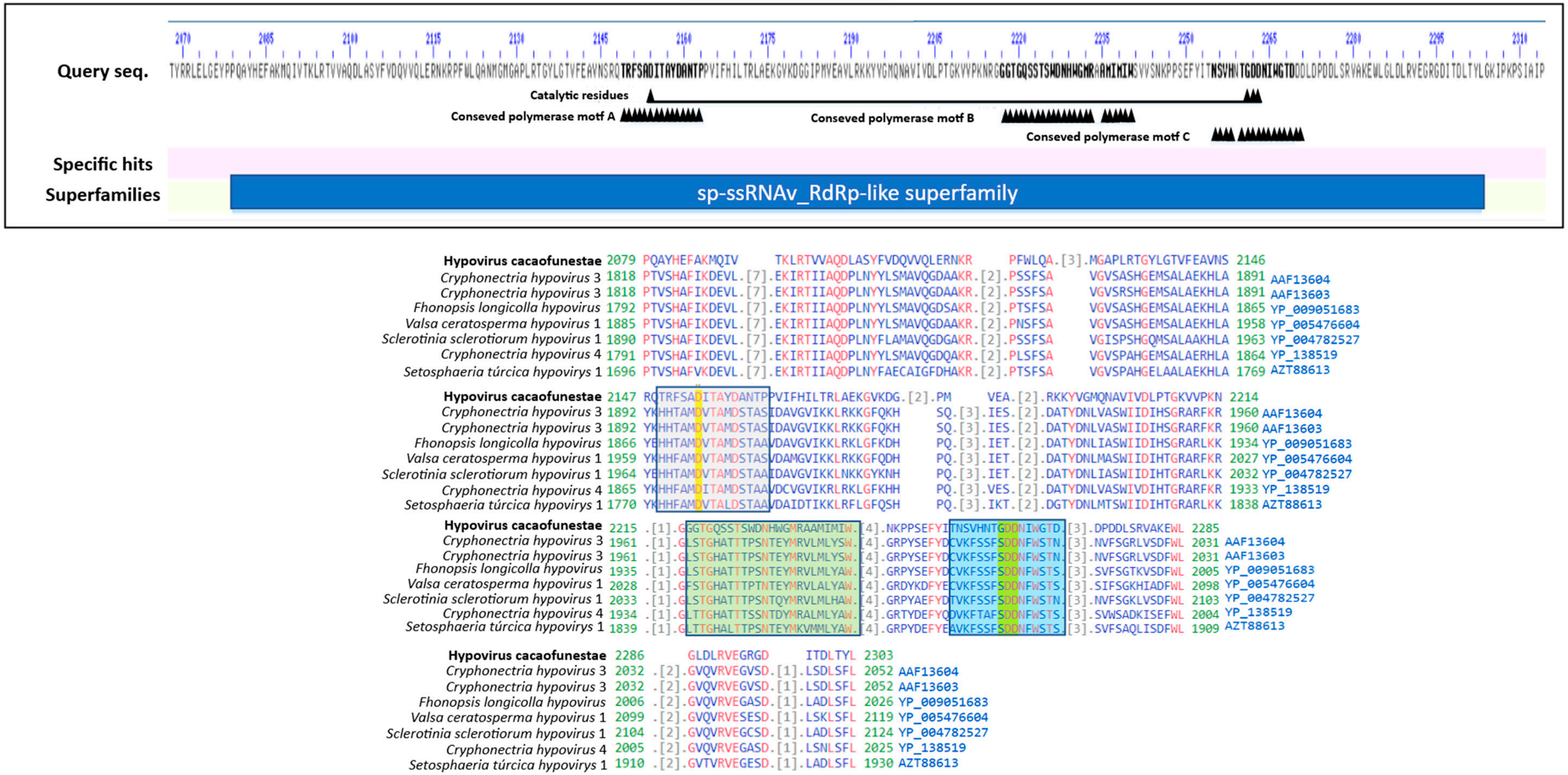

Figure S2. Conserved motifs and catalytic site in the Hypovirus cacaofunestae viral contig. A. Superfamily cl40470 similar to ps-ssRNAv_RdRp located in the interval 2079-2303, conserved polymerase motif A, located at position 2149-2163, conserved polymerase motif B, located at position 2217-2233 and 2235-2240, conserved polymerase motif C, located at position 2255-2258 and 2259-2269 B. Species of the family *Hypoviridae* with similarity of motifs conserved with HVc, Catalytic site (Yellow), Motif A (Light Grey), Motif B (Light Green), Motif C (Lemon Green).

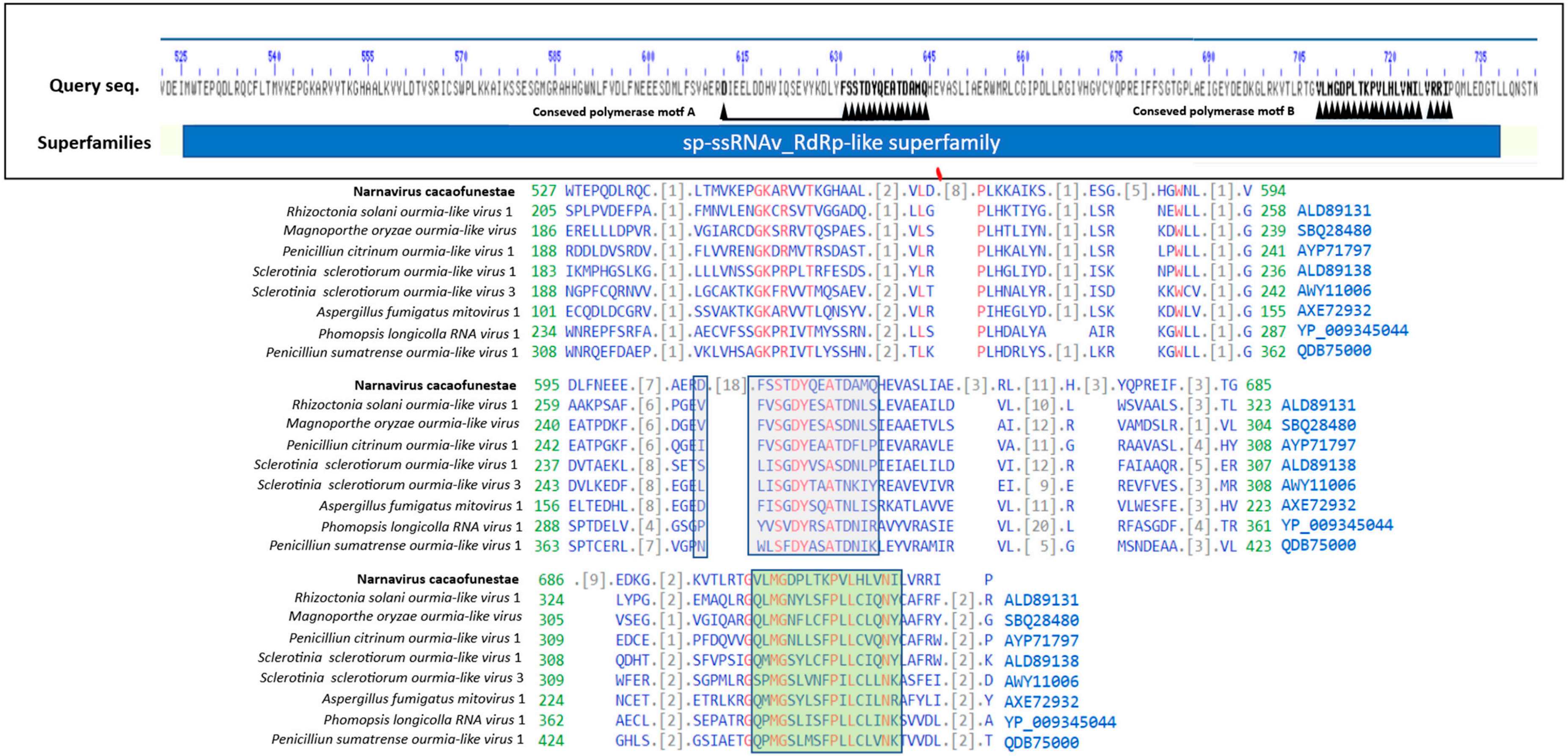

Figure S3. Conserved motifs and catalytic site in the Narnavirus cacaofunestae viral contig. A. Superfamily cl40470 similar to ps-ssRNAv_RdRp located in the interval 527-736, conserved polymerase motif A, located at positions 612 and 631-644 , conserved polymerase motif B, located at positions 698-724 and 726-729. B. Species of the family Botourmiaviridae and Mitoviridae with similarity of motifs conserved with NVf, Motif A (light grey), Motif B (light green).

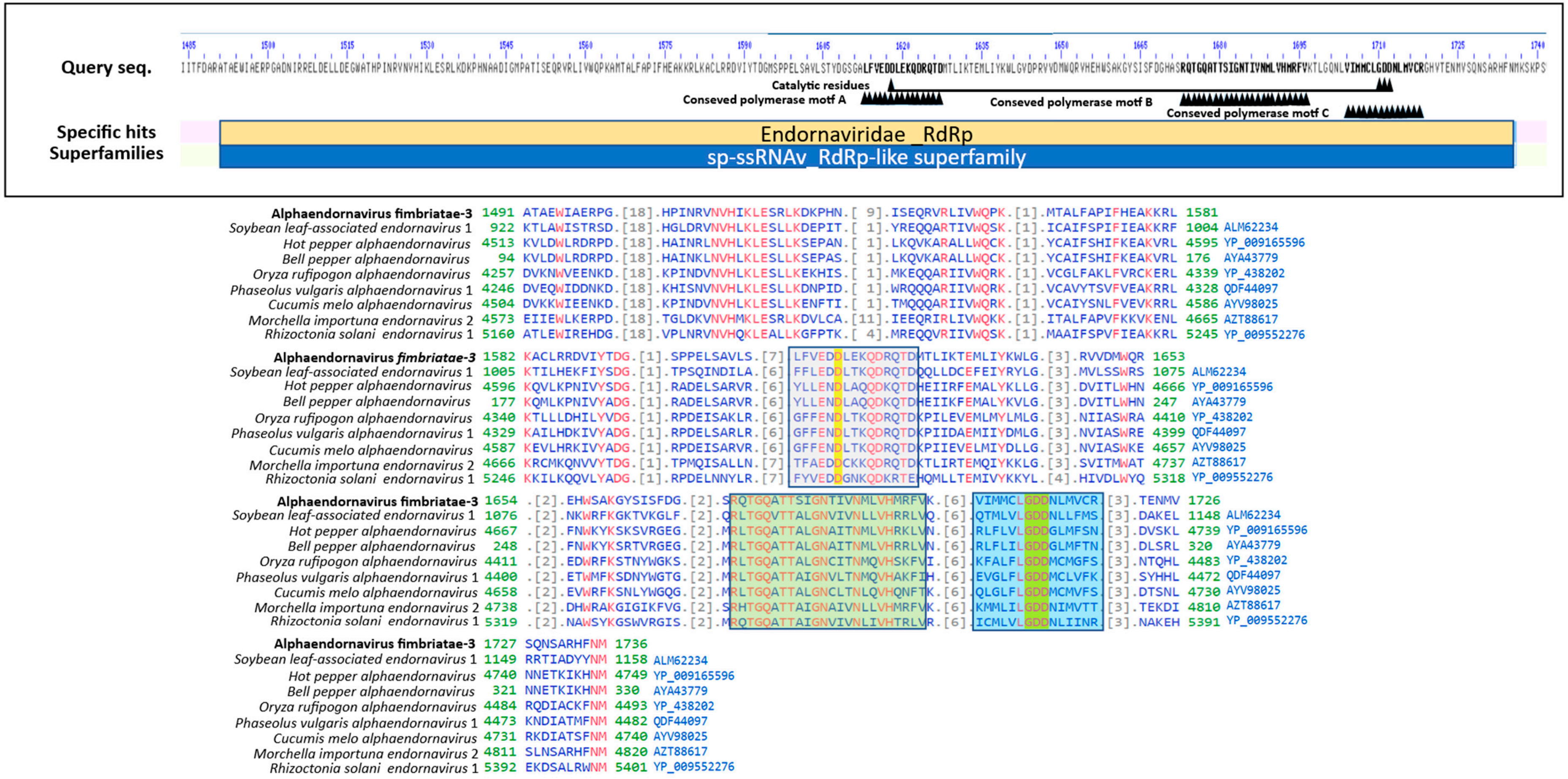

Figure S4. Conserved motifs and catalytic site in the Alphaendornavirus fimbriatae-3 viral contig. A. Superfamily cl40470 similar to ps-ssRNAv_RdRp located in the interval 1491-1736, conserved polymerase motif A, located at position 1613-1627, conserved polymerase motif B, located at position 1673-1696, conserved polymerase motif C, located at position 1704-1718 B. Species of the family *Endornaviridae* with similarity of motifs conserved with AEVf-3, Catalytic site (Yellow), Motif A (Light Grey), Motif B (Light Green), Motif C (Blue).

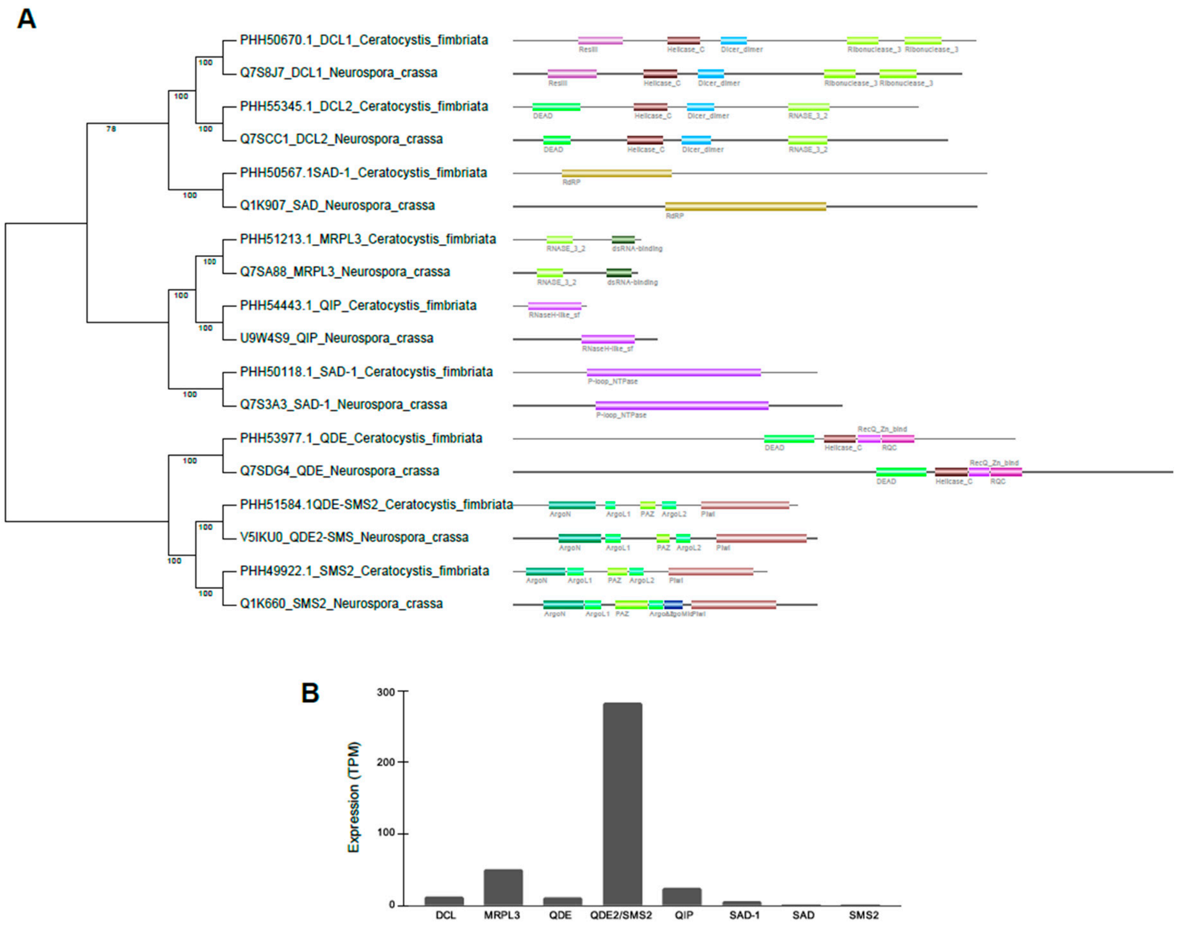

Figure S5. Characterization of RNAi-related genes in *Ceratocystis fimbriata*. (A) Dendrogram based on sequence similarity. (B) Evaluation of RNAi-related genes transcriptional activity. Quantification was normalized by Transcripts Per Million (TPM).
